# A77 THE INFLUENCE OF IRON SUPPLEMENTATION IN EARLY LIFE ON COLORECTAL CANCER PROGRESSION

**DOI:** 10.1093/jcag/gwae059.077

**Published:** 2025-02-10

**Authors:** C Gerkins, T Cuisiniere, T Maumy, C McCartney, G Fragoso, A Calve, M M Santos

**Affiliations:** Nutrition and Microbiome Laboratory, Institut du cancer de Montréal, Centre de recherche du Centre hospitalier de l’Université de Montréal (CRCHUM), Montreal, QC, Canada; Nutrition and Microbiome Laboratory, Institut du cancer de Montréal, Centre de recherche du Centre hospitalier de l’Université de Montréal (CRCHUM), Montreal, QC, Canada; Nutrition and Microbiome Laboratory, Institut du cancer de Montréal, Centre de recherche du Centre hospitalier de l’Université de Montréal (CRCHUM), Montreal, QC, Canada; Nutrition and Microbiome Laboratory, Institut du cancer de Montréal, Centre de recherche du Centre hospitalier de l’Université de Montréal (CRCHUM), Montreal, QC, Canada; Nutrition and Microbiome Laboratory, Institut du cancer de Montréal, Centre de recherche du Centre hospitalier de l’Université de Montréal (CRCHUM), Montreal, QC, Canada; Nutrition and Microbiome Laboratory, Institut du cancer de Montréal, Centre de recherche du Centre hospitalier de l’Université de Montréal (CRCHUM), Montreal, QC, Canada; Department of Medicine, Faculty of Medicine, Université de Montréal, Montreal, QC, Canada

## Abstract

**Background:**

During the first years of life, the immune system develops simultaneously with the gut microbiota, and it has been previously shown that perturbations to gut microbiota development can lead to altered immune phenotypes later in life. One factor that may influence the gut microbiota in early life, and therefore immune development, is iron supplementation.

**Aims:**

We aim to evaluate how iron supplementation in early life affects immune system development and therefore cancer progression in adulthood.

**Methods:**

BALB/c mice were given either an iron sufficient or an iron excess diet beginning at weaning until adulthood at 8 weeks of age. All mice were then placed on the iron sufficient diet for two weeks prior to beginning the experimental model in order to establish the effect of iron only during the early life period. Mice were then injected subcutaneously with the murine colon carcinoma cell line CT-26.

**Results:**

Mice that recieved iron supplementation in early life had significantly higher levels of iron in both the liver and spleen, indicating that this diet was successful in increasing iron levels. Furthermore, mice given iron supplementation had significantly more sebaceous glands per mm ear tissue than mice given the iron sufficient diet. Sebaceous glands are structures in the skin important for coordination of the innate immune response and regulation of the skin microbiota. We next measured several cytokines by ELISA. Mice supplemented with iron had significantly lower levels of interleukin (IL)-4, IL-10, and interferon (IFN)-γ.

**Conclusions:**

Considering that mice given the iron excess diet had significantly altered skin immune structures and significantly decreased basal cytokine levels in ear tissue, we conclude that iron supplementation in early life modulates immune system development. Next, we will evaluate how iron supplementation in early life affects colorectal cancer development and progression.

**Funding Agencies:**

CIHRFRQS, NSERC

